# Childhood socioeconomic status, comorbidity of chronic kidney disease risk factors, and kidney function among adults in the midlife in the United States (MIDUS) study

**DOI:** 10.1186/s12882-020-01846-1

**Published:** 2020-05-19

**Authors:** Agus Surachman, Jonathan Daw, Bethany C. Bray, Lacy M. Alexander, Christopher L. Coe, David M. Almeida

**Affiliations:** 1 Department of Human Development and Family Studies/ Center for Healthy Aging, The Pennsylvania State University, 405 Biobehavioral Health (BBH) Building, University Park, PA 16802 USA; 2 Center for Healthy Aging, The Pennsylvania State University, University Park, PA USA; 3 Department of Sociology, The Pennsylvania State University, University Park, PA USA; 4 The Methodology Center, The Pennsylvania State University, University Park, PA USA; 5 Center for Dissemination and Implementation Science, UIC, Chicago, IL USA; 6 Department of Human Development and Family Studies, The Pennsylvania State University, University Park, PA USA; 7 Department of Psychology, University of Wisconsin – Madison, Madison, WI USA; 8 Harlow Center for Biological Psychology, University of Wisconsin – Madison, Madison, WI USA

**Keywords:** Kidney disease, Early adversity, Diabetes, Obesity, Hypertension, Socioeconomic status

## Abstract

**Background:**

There is a lack of empirical effort that systematically investigates the clustering of comorbidity among known risk factors (obesity, hypertension, diabetes, hypercholesterolemia, and elevated inflammation) of chronic kidney disease (CKD) and how different types of comorbidity may link differently to kidney function among healthy adult samples. This study modeled the clustering of comorbidity among risk factors, examined the association between the clustering of risk factors and kidney function, and tested whether the clustering of risk factors was associated with childhood SES.

**Methods:**

The data were from 2118 participants (ages 25–84) in the Midlife in the United States (MIDUS) Study. Risk factors included obesity, elevated blood pressure (BP), high total cholesterol levels, poor glucose control, and increased inflammatory activity. Glomerular filtration rate (eGFR) was estimated from serum creatinine, calculated with the CKD-EPI formula. The clustering of comorbidity among risk factors and its association with kidney function and childhood SES were examined using latent class analysis (LCA).

**Results:**

A five-class model was optimal: (1) ***Low Risk*** (class size = 36.40%; low probability of all risk factors), (2) ***Obese*** (16.42%; high probability of large BMI and abdominally obese), (3) ***Obese and Elevated BP*** (13.37%; high probability of being obese and having elevated BP), (4) ***Non-Obese but Elevated BP*** (14.95%; high probability of having elevated BP, hypercholesterolemia, and elevated inflammation), and (5) ***High Risk*** (18.86%; high probability for all risk factors). Obesity was associated with kidney hyperfiltration, while comorbidity between obesity and hypertension was linked to compromised kidney filtration. As expected, the High Risk class showed the highest probability of having eGFR < 60 ml/min/1.73 m^2^ (*P* = .12; 95%*CI* = .09–.17). Finally, higher childhood SES was associated with reduced probability of being in the High Risk rather than Low Risk class (β = − 0.20, *SE* = 0.07, OR [95%*CI*] = 0.82 [0.71–0.95]).

**Conclusion:**

These results highlight the importance of considering the impact of childhood SES on risk factors known to be associated with CKD.

## Background

The risk for Chronic Kidney Disease (CKD) is heightened among individuals with risk factors such as obesity, hypertension, diabetes, hypercholesterolemia, and elevated inflammation [1–5]. Comorbidity among these risk factors is common and often leads to a faster progression to CKD [2, 6]. Different characteristics of comorbidity among these risk factors may be linked to a different state of kidney functioning. For example, in the early stage of obesity when hypertension is absent, obese individuals show elevated kidney filtration as a sign of an early adaptive process to hemodynamic changes due to obesity [6, 7]. On the other hand, comorbidity between obesity and hypertension leads to progressive damage to kidney structure and thus incremental declines in kidney function [6–8]. Multiple pathways link obesity to hypertension and CKD, including hypercholesterolemia, hyperglycemia, and inflammation [6]. However, there is still limited empirical research that systematically investigates the clustering of comorbidity among these risk factors and how different aspects of comorbidity contribute to declining kidney functioning among healthy adult samples. This knowledge is important for prevention efforts to reduce the risk of progression to CKD [2].

### Contextualizing the clustering of risk factors

Understanding the social factors associated with the development and clustering of risk factors is integral to preventing CKD. Socioeconomic status (SES) is often associated with the known risk factors. SES is a general term for a group of valued resources, comprising both economic or material resources and also prestige or social status [9]. The burden of CKD is not evenly distributed in the population as the prevalence of CKD is higher among individuals from lower levels SES [10]. Socioeconomic disparities in CKD are mediated by each of the major risk factors including obesity, hypertension, diabetes, hypercholesterolemia, and inflammation [5, 11]. However, there is a lack of empirical studies that directly examined the association between SES and the clustering among CKD risk factors.

Furthermore, SES is a dynamic concept, and its influence on health may span across different developmental stages across the life course [12, 13]. Throughout the life course, there are at least three important periods in which one’s SES may have a significant impact on health [14–16]: 1) SES during childhood as determined by one’s parental SES, 2) formal education attainment throughout early adulthood that may influence one’s future social and economic prospect across adulthood, and 3) current SES in adulthood, reflected by income level and social status. Recently, there have been more interests in documenting the influence of childhood SES on the development of kidney disease and its risk factors [13, 17, 18]. Multiple studies have documented a significant influence of socioeconomic adversity during childhood on the emergence and presentation of disease in adulthood [19, 20]. Our analysis focused on the potential association between childhood SES and risk factors associated with CKD. Childhood SES may initiate the developmental trajectory toward adult CKD by influencing the development and clustering of the risk factors. Previous studies have shown that low childhood SES, independent of education level and current level of SES (e.g., income), was associated with a higher likelihood of obesity [21, 22], elevated BP [23, 24], diabetes [25, 26], and increased inflammatory physiology [27, 28] later in adulthood. However, our understanding is still limited when it comes to the association between childhood SES and the comorbidity of these risk factors and how the comorbidity is manifested by decreased kidney function in middle and later adulthood.

## Method

### Participants and procedure

Data for this study are from the Midlife in the United States (MIDUS) study, a national study of health and well-being involving a national probability sample of middle and older adults from the 48 continental states [29]. MIDUS started in 1995–1996 (MIDUS 1), and included 7108 adults, ages 25–74, recruited through random digit dialing (RDD). They completed a baseline telephone interview and then the majority (89% of the total sample) also completed self-administered questionnaires (SAQ). The longitudinal follow-up of MIDUS was conducted in 2004–2006 (MIDUS 2), which included 4963 longitudinal participants. Similar to MIDUS 1, all the participants in MIDUS-2 completed a baseline telephone interview, during which 81% also completed the SAQ. To increase the racial diversity of the MIDUS 2 sample, a supplemental sample consisting of majority Black adults who were recruited from Milwaukee County, WI (*n* = 592). Similar to the national sample in MIDUS 2, all the Milwaukee participants also completed a baseline interview and the majority of them (89% of the total sample) completed the SAQ. A new protocol of specimen collection and biomarker testing was introduced during MIDUS 2. Participation in the baseline interview and completion of SAQ was the eligibility criteria for participation in the biomarker protocol. In addition, respondents were eligible to participate in the biomarker assessment if their existing health information indicated an ability to travel to the clinic without unwarranted risk to the respondent or project staff [30]. In MIDUS 2, 1255 participants, both from the national sample and the Milwaukee supplemental sample, completed the biomarker assessment.

In 2012–2016, a new national probability sample (*n* = 3577) that matched the original MIDUS sample (MIDUS 1) in terms of their sociodemographic characteristics was recruited to participate in the MIDUS Refresher study (MIDUS R). This sample was recruited to replenish the number of middle-aged adults given that the initial cohort was now older [14, 31]. Similar to MIDUS 1 and 2, participants in MIDUS R were recruited through RDD and all completed the baseline telephone interview. The majority in MIDUS R (73%) also completed the SAQ. Similar to MIDUS 2, a supplemental sample was also recruited from Milwaukee County in order to increase the racial diversity of the sample in MIDUS R (*n* = 508). Among the supplemental sample in MIDUS R, 299 participants (59% of the in-person interview participants) completed the SAQ. MIDUS R also includes biomarker assessment protocol, with the same eligibility requirement as in MIDUS 2 (completed the baseline survey and SAQ and able to travel to the clinic). In MIDUS R, 863 participants (randomly selected from the national sample and the Milwaukee supplemental sample) completed the biomarker assessment.

For the current analysis, data from all participants who completed the biomarker assessments in MIDUS 2 and MIDUS R were included (*N* = 2118; ages 25–84; 54.9% female; 73.7% non-Hispanic White). The biomarker assessment protocol in MIDUS 2 and MIDUS R was identical. Participants were invited to stay overnight at one of the three regional clinical research units (CRUs; West Coast, Midwest, and East Coast). The selection of the CRU for each participant was based on the one that involved the least travel burden. Blood and urine samples were collected during the stay. Participants provided informed consent to participate in both the baseline survey and the biomarker assessment. Additional information regarding biomarker assessment in the MIDUS study can be found elsewhere [30].

### Measures

#### Risk factors

Seven known risk factors for CKD were included in this analysis: (1) elevated blood pressure/ BP (mean of second and third blood pressure test: systolic and diastolic blood pressure ≥ 140/90 mmHg or self-reported diagnosis of hypertension by a physician; (2) elevated glycosylated hemoglobin (HbA1c ≥ 6.5%) or high fasting blood glucose (≥ 126 mg/dL) or self-reported diagnosis of type 2 diabetes by a physician; (3) obese (BMI ≥ 30 kg/m2); (4) abdominal obesity (waist circumference ≥ 88 cm for women and ≥ 102 cm for men); (5) hypercholesterolemia (total serum cholesterol ≥200 mg/dL); (6) elevated c-reactive protein (CRP ≥ the third quartile); and (7) elevated interleukin 6 (IL6 ≥ the third quartile).

#### Kidney function

The estimated glomerular filtration rate (eGFR) was estimated from serum creatinine using the CKD-EPI formula [32]. Serum creatinine was assayed from overnight fasted blood collected at the three CRUs using Roche Cobas Analyzer (Meriter Clinical Lab, Madison, WI; inter-assay coefficient of variability = 2.08%). The overall mean of eGFR was 91.2 mL/min/1.73 m^2^ (*SD* = 19.2 mL/min/1.73 m^2^). For further analysis, eGFR was transformed into a binary variable based on the clinical indicator of Stage 3 CKD (1 = eGFR lower than 60 ml/min/1.73 m2, *n* = 107 [5.1%]; 0 = the rest of participants).

#### Childhood SES

Childhood SES was the total score from three indicators, including (1) father (or mother in case of missing data) highest level of education (0 = < high school, 1 = graduated from high school/GED, 2 = some college or higher); (2) whether the family of origin received welfare (0 = all the time/most of the time, 1 = some of the time/a little of the, 2 = never in welfare); and (3) financial level growing up (0 = a lot/somewhat/a little worse off than average family, 1 = same as average family, 2 = a lot/somewhat, a little better off than average family). The mean childhood SES score was 3.91 (*SD* = 1.45; range = 0–6). This set of childhood SES measure is a significant predictor of health outcomes in adulthood, such as allostatic load, chronic disease, and diabetes [14, 15, 33].

#### Covariates

Covariates in the analysis include participant’s highest formal education level (0 = no high school diploma/ GED; 1 = graduated from high school and higher) and current/ adult SES. Adult SES was the total score based on five indicators [14, 15, 33], including: (1) household-size adjusted income to poverty ratio (0 = < 150%, 1 = ≥ 150% - < 300%, 2 = ≥ 300%); (2) current financial situation (0 = worse, 1 = average, 2 = best); (3) availability of money to meet basic needs (0 = not enough money, 1 = just enough money, 2 = more money than need); and (4) difficulty level paying bills (0 = very/somewhat difficult, 1 = not very difficult, 2 = not at all difficult). This set of adult SES variables has been shown to be a significant predictor of multiple health outcomes in adulthood, including allostatic load [15], inflammatory biomarkers [16], bone mineral density [34], and physical activity [35]. Sociodemographic variables were also incorporated as covariates, including age (years), gender (female = 0, male = 1), and race/ ethnicity (minority = 0, non-Hispanic White = 1).

### Statistical analysis

**Fig. 1:**
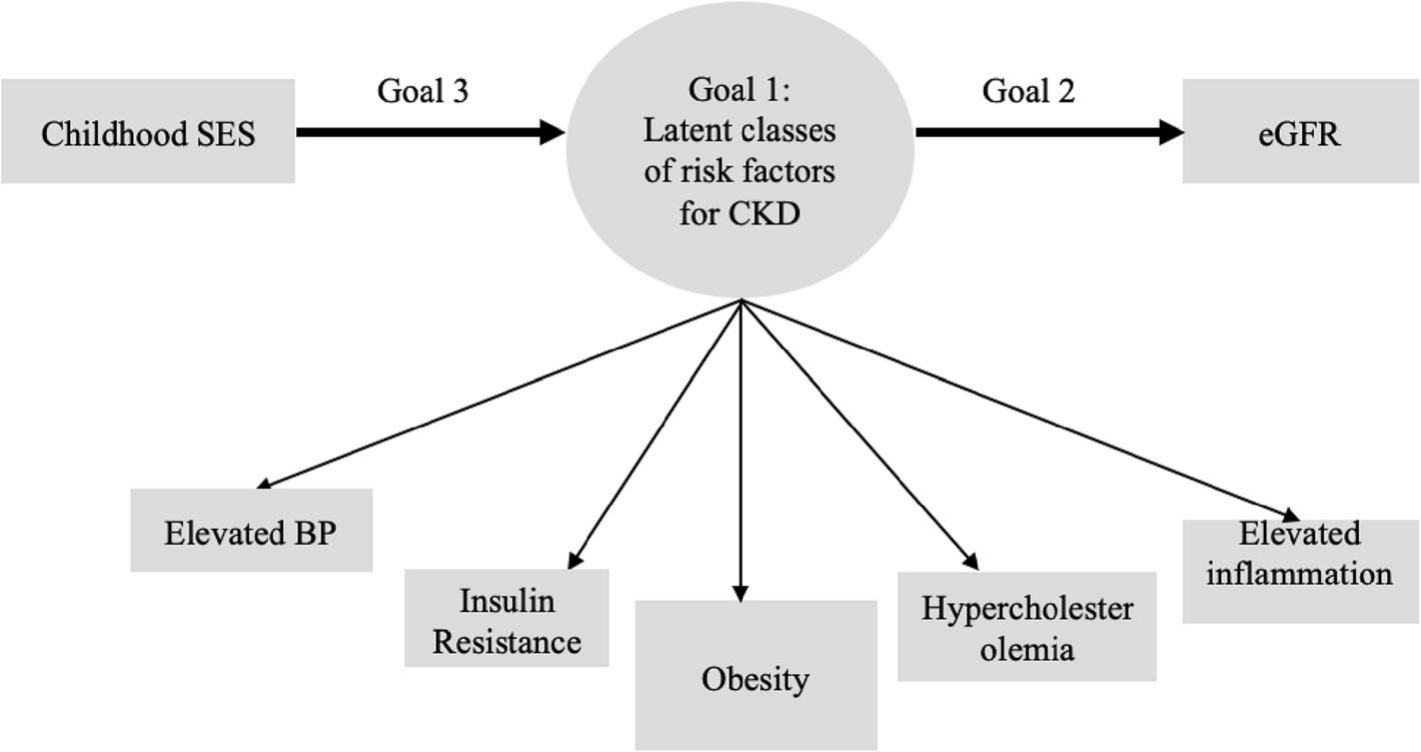
Visual representation of the hypothesis tested in this paper. First, we identified the heterogeneity of comorbidity in risk factors using latent class analysis (LCA). Second, we tested whether different characteristics of comorbidity in risk factors have different impact on kidney function. Third, we examined whether childhood SES, independent of education and adult SES, predicted latent classes of comorbidity in risk factors

The following analysis had three primary aims (Fig. 1): (1) to model the heterogeneity of comorbidity among CKD risk factors by examining the clustering of risk factors associated with age-related declines in kidney function among middle-aged and older adults; (2) to empirically test whether the clustering of comorbidity among CKD risk factors was associated with a different state of kidney functioning; and (3) to contextualize the different clustering of CKD risk factors by testing whether childhood SES, controlling for education, adult SES, age, gender, and race, was associated with the clustering of risk factors. Latent class analysis (LCA) was employed to address these research questions. A person-centered analysis such as LCA provides objective and parsimonious solutions regarding the variation in the clustering of risk factors, its impact on kidney function, and prediction by childhood SES. The analysis was divided into three steps. First, we identified the heterogeneity of the comorbidity among risk factors. Second, the association between latent classes of risk factors and kidney function was examined. Third, we tested the evidence of whether childhood SES was associated with the heterogeneity of comorbidity among CKD risk factors by utilizing a model-based approach LCA.

#### Step 1: examination of the heterogeneity of comorbidity among risk factors

The selection of the optimally fitting model was based on model fit statistics and selection criteria, parsimony principle, as well as theoretical interpretability. Model fit statistics and selection criteria included the Akaike information criterion (AIC), Bayesian information criterion (BIC), sample-size adjusted BIC (a-BIC), entropy, Bozdogan’s consistent AIC (CAIC), and bootstrapped likelihood ratio test (BLRT). A better fit model is indicated by lower values for the AIC, BIC, and a-BIC. In addition, higher values for entropy indicated higher classification utility. Finally, significant *p*-values of the bootstrapped likelihood ratio test indicated an improved model fit compared to models with one fewer class. Two, three, four, five, and six latent classes LCAs were compared to select the best fitting model. Model identification was conducted by using 1000 sets of random starting values; all models were estimated using PROC LCA on SAS [36]. Two sets of parameters are of most interest from the best fitting model. The first set is the latent class membership probabilities, which indicate the distribution of the classes in the population. The second set is the item-response probabilities, which indicate the probabilities of providing certain responses to observed variables conditional on class membership [36]. These two sets of parameters are used to label and interpret the classes. The analysis was conducted using PROC LCA on SAS [36].

#### Step 2: testing the association between the heterogeneity of comorbidity among risk factors and kidney function

In the second step, we examined whether latent classes of CKD risk factors were predictive of eGFR. We used eGFR as both a continuous and binary variable (1 = < 60 ml/min/1.73 m^2^, 0 = ≥ 60 ml/min/1.73 m^2^). For the analysis with eGFR as a continuous variable, the outcome from the analysis was the expected mean of eGFR for each latent class of CKD risk factors. When predicting eGFR as a binary variable, the outcome of the analysis indicated the probability of having eGFR lower than 60 ml/min/1.73 m^2^ for each latent class. We utilized LCA with a distal outcome to test this hypothesis using the BCH approach [37]. The LCA with a distal outcome was executed using LCA_Distal_BCH SAS macro [38].

#### Step 3: examining the association between childhood SES and the heterogeneity of risk factors

In the final step of the analysis, we examined whether childhood SES was associated with class membership, after controlling for education level, current SES, age, gender, and race. We tested the hypothesis by utilizing model-based approach LCA with covariates [36], in which childhood SES (score) was utilized to predict the probability of belonging to a certain latent class of risk factors comorbidity (relative to the reference class), controlling for the covariates. The results were presented as the odds ratios of belonging to a certain class compared to the reference class. The model-based approach LCA was conducted using PROC LCA on SAS [36].

#### Missing data

Parameters in PROC LCA are estimated by maximum likelihood using an EM (expectation-maximization) procedure [36]. This procedure accommodates missing data when identifying the latent class indicators, assuming that data missing at random (MAR) [36].

## Results

### Descriptive statistics

Descriptive statistics for the participants are presented in Table 1. More than half of the participants in this study met the criteria of having elevated BP, being abdominally obese, and hypercholesterolemic. Slightly more than half of the participants had a healthy level of kidney function (eGFR ≥90 ml/min/1.73 m^2^). The proportion of participants with eGFR < 60 ml/min/1.73 m^2^ was around 5%. In terms of childhood SES, around one-third of the participants reported their parents did not finish high school. Similarly, almost one-third of the participants also reported that their families’ financial status during their childhood was low compared to other families around them.

**Table 1:** Descriptive Statistics for Class Indicators, Covariates, and Outcome (*N* = 2118)

Variables	MIDUS 2 (***n*** = 1255)	MIDUS R (***n*** = 863)	Overall (***N*** = 2118	***p***-value ^a^
**Risk Factors**				
Elevated blood pressure (Systolic/diastolic ≥140/90 or diagnosed by physician; %)	52.7	48.6	51.0	*ns*
Insulin resistance (HbA1c ≥ 6.5% or blood fasting glucose ≥126 mg/dL or diagnosed by physician; %)	20.1	17.4	19.0	*ns*
Obese (BMI ≥ 30 Kg/m^2^; %)	41.2	45.2	42.8	*ns*
Abdominally Obese (waist circumference ≥ 88 cm for women and ≥ 102 cm for men; %)	55.6	54.7	55.2	*ns*
Hypercholesterolemic (total serum cholesterol ≥200 mg/dL or diagnosed by physician; %)	60.1	55.6	58.3	< .05
Elevated IL6 (%)	32.2	24.8	29.2	< .01
Elevated CRP (%)	31.2	24.7	28.5	< .001
**Sociodemographic Correlates**				
**Childhood SES**				
Parental education less than HS/GED (%)	42.2	24.2	34.9	< .001
Family of origin received welfare (%)	2.9	4.4	3.5	< .05
Low subjective financial status (%)	27.4	33.6	29.9	< .001
Mean total score of childhood SES (*SD;* min-max)	3.80 (1.43; 0–6)	4.08 (1.46; 0–6)	3.91 (1.45; 0–6)	< .001
**Covariates**				
Female (%)	56.8	52.1	54.9	< .05
Mean age (*SD;* min-max)	54.5 (11.7; 34–84)	50.8 (13.4; 25–76)	53.0 (12.6; 25–84)	< .001
Non-Hispanic White (%)	77.2	68.6	73.7	< .001
Education less than HS/GED (%)	27.9	17.3	23.6	< .001
Mean total score adult SES (*SD;* min-max)	4.73 (2.28; 0–8)	4.39 (2.38; 0–8)	4.59 (2.33; 0–8)	< .01
**Kidney Function (*M*,*SD*, range, %)**				
Mean eGFR (*SD*, min-max)	90.4 (19.6; 3.7–150.7)	92.4 (18.5; 20.4–139.5)	91.2 (19.2; 3–7-150.7)	< .05
eGFR < 60 ml/min/1.73 m^2^	5.6	4.3	5.1	< .05
eGFR 60–89 ml/min/1.73 m^2^	42.6	37.5	40.6	
eGFR ≥90 ml/min/1.73 m^2^	50.9	56.9	53.4	

*Note*: MIDUS 2 = MIDUS wave 2, MIDUS R = MIDUS Refresher; *M* = mean, *SD* = standard deviation; a = *p*-values from difference tests between MIDUS 2 and MIDUS R

### The clustering of risk factors

Models with 1–6 classes were tested; the 4-class model had the lowest level of BIC and CAIC (see Supplemental Table 1 for information regarding model fit statistics and selection criteria). However, the 5-class model showed a better fit based on AIC and aBIC. The bootstrapped likelihood ratio test showed that the 5-class model was the last model with a significant *p*-value. Thus, moving from the 5-class model to the 6-class model did not significantly improve the model fit. Entropy ranged from .59 (2-class model) to .76 (6-class model), with values for larger classes in the mid-to-upper .70s. Therefore, we considered models with 4 or 5 classes. Closer examination indicated that an additional class in the 5-class model had a non-repetitive, meaningful, and interpretable class. Thus, we selected the 5-class model for theoretical explanation and distal outcome analysis.

Table 2 provides information about latent class membership probabilities and item-response probabilities for the 5-class model. Class 1 was labeled ***Low Risk*** (class size = 36.40), characterized by low probabilities for all the CKD risk factors. Class 2 was labeled as ***Obese*** (16.42%) given that this group of individuals had a high probability of being obese. Class 3 was identified as ***Obese and Elevated BP*** (13.37%) because these adults had elevated probabilities for having elevated BP with hypercholesterolemia in addition to being obese. Class 4 was characterized as ***Non-Obese but Elevated BP*** (14.95%). They were distinguished by high probabilities for elevated BP, hypercholesterolemia, and elevated CRP, but without indications of extreme adiposity. The final class was delineated as ***High Risk*** (18.86%), characterized by high probabilities for all identified risk factors.

**Table 2:** Latent Class Membership and Item-Response Probabilities

Indicator	Class 1: Low Risk (36.40%)	Class 2: Obese (16.42%)	Class 3: Obese and Elevated BP (13.37%)	Class 4: Non-Obese but Elevated BP (14.95%)	Class 5: High Risk (18.86%)
Elevated blood pressure (Systolic/diastolic ≥140/90 or diagnosed by physician; %)	.28	.43	**.70**	**.60**	**.86**
Insulin resistance (HbA1c ≥ 6.5% or blood fasting glucose ≥126 mg/dL or diagnosed by physician; %)	.07	.00	.28	.17	**.55**
Obese (BMI ≥ 30 Kg/m^2^; %)	.02	**.93**	**.68**	.00	**.94**
Abdominally Obese (waist circumference ≥ 88 cm for women and ≥ 102 cm for men; %)	.10	**.93**	**.93**	.37	**.99**
Hypercholesterolemic (total serum cholesterol ≥200 mg/dL or diagnosed by physician; %)	.47	.49	**.78**	**.64**	**.71**
Elevated IL6 (≥ the third quartile)	.07	.44	.00	.41	**.68**
Elevated CRP (≥ the third quartile)	.02	.28	.00	**.66**	**.7**4

*Note*: boldface type indicates high probability

### The association between latent classes of comorbidity among risk factors and kidney function

**Fig. 2:**
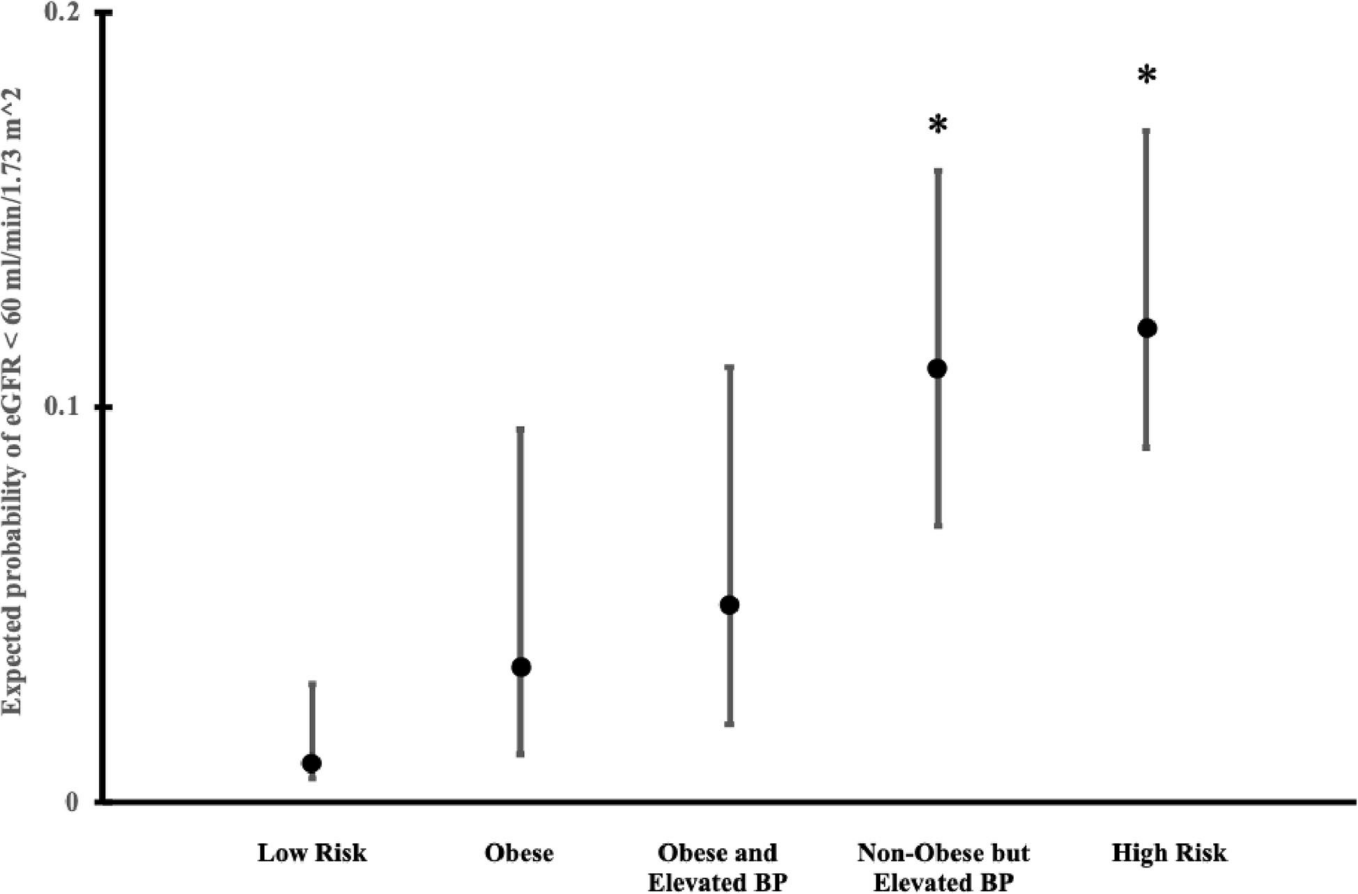
Expected probability with 95% *CI* for having eGFR < 60 ml/min/1.73 m^2^ based on classes of CKD risk factors comorbidity; omnibus test: χ^2^ (4) = 23.66, *p* < .001; *: significantly higher than the Low Risk class (*p* < .05; Bonferroni correction applied for multiple comparisons in pairwise comparison tests)

The distal outcome analysis indicated that class membership was associated with eGFR (Wald χ^2^ (4) = 44.04, *p* < .001). The Obese class had the highest expected mean of eGFR (99.77 ml/min/1.73 m^2^, *SE* = 1.70 ml/min/1.73 m^2^), followed by the Low Risk (*M* = 93.05, *SE* = 0.69), the High Risk (*M* = 88.97, *SE* = 1.53), the Non-Obese but Elevated BP (*M* = 85.45, *SE* = 1.6), and the Obese and Elevated BP (*M* = 85.43, *SE* = 1.71). The association between class membership and kidney function was more apparent when considering eGFR as a binary variable (0 = eGFR > = 60 mL/min/m^2^, 1 = eGFR < 60 mL/min/m^2^; Fig. 2). The results showed that class membership was associated with different probability of having eGFR < 60 ml/min/1.73 m^2^ (Wald χ^2^ (4) = 23.66, *p* < .001). The High Risk class had the highest expected probability (*P* = .12; 95% *CI* = .09–.17), while the Low Risk evinced the lowest expected probability (*P* = .01; 95% *CI* = .006–.03) of having eGFR < 60 ml/min/1.73 m^2^. The expected probabilities for the rest of the classes are as follow (lower to higher): Obese (*P* = .03; 95% *CI* = .01–.09), Obese and Elevated BP (*P* = .05; 95% *CI* = .02–.11), and Non-Obese but Elevated BP (*P* = .11; 95% *CI* = .07–.16). Pairwise comparisons indicated that the expected probability of having eGFR < 60 ml/min/1.73 m^2^ for the High Risk (Wald χ^2^ (1) = 20.81, *p* < .050) and Non-Obese but Elevated BP (Wald χ^2^ (1) = 13.43, *p* < .050) class was significantly higher than the probability for the Low Risk class (Bonferroni correction applied for multiple comparison).

### The association between childhood SES and the latent classes of comorbidity among risk factors

Childhood SES was significantly associated with latent class membership of risk factors, even after controlling for covariates (Table 3; χ^2^ [4] = 15.28, *p* < .01). Higher childhood SES was significantly associated with lower probability of being in the Obese and Elevated BP class (β = − 0.22, *SE* = 0.07, OR [95%*CI*] = 0.81 [0.70–0.93]), as opposed to being in the Low Risk class. Furthermore, higher childhood SES was also significantly associated with lower probability of being in the High Risk (β = − 0.20, *SE* = 0.07, OR [95%*CI*] = 0.82 [0.71–0.95]) rather than the Low Risk class. For every 1 point higher in childhood SES score, participants were 19% less likely to be in the Obese and Elevated BP class and 18% less likely to be in the High Risk class, as opposed to being in the healthier Low Risk class. However, childhood SES was not significantly associated with the probability of membership in the Obese (β = 0.09, *SE* = 0.09, OR [95%*CI*] = 1.09 [0.91–1.31]) and Non-Obese but Elevated BP (β = − 0.10, *SE* = 0.08, OR [95%*CI*] = 0.91 [0.77–1.06]) classes relative to the Low Risk class.

**Table 3:** Childhood SES and probability of being in the non-healthy classes rather than the Low Risk class (*N* = 2118)

Predictors	Latent Classes								
	χ^2^ (*df* = 4)	Obese (16.42%)		Obese and Elevated BP (13.37%)		Non-Obese but Elevated BP (14.95%)		High Risk (18.86%)	
		β (SE)	OR [95%CI]	β (SE)	OR [95%CI]	β (SE)	OR [95%CI]	β (SE)	OR [95%CI]
Childhood SES (score)	15.28 ^**^	0.09 (0.09)	1.09 [0.91–1.31]	**−0.22 (0.07)**	0.81 [0.70–0.93]^*^	−0.10 (0.08)	0.91 [0.77–1.06]	**− 0.20 (0.07)**	0.82 [0.71–0.95]^*^
Education (0 = no HS/GED; 1 = HS/GED or higher)	12.27 ^*^	− 0.67 (0.35)	0.51 [0.26–1.01]	−0.34 (0.30)	0.71 [0.40–1.27]	− 0.47 (0.30)	0.63 [0.35–1.12]	**−0.78 (0.27)**	0.46 [0.27–0.78]^*^
Adult SES (score)	72.69 ^***^	**−0.16 (0.06)**	0.86 [0.77–0.95]^*^	**−0.23 (0.06)**	0.79 [0.70–0.90]^*^	**− 0.32 (0.06)**	0.72 [0.65–0.81]^*^	**−0.40 (0.05)**	0.67 [0.61–0.75]^*^
Age (years)	328.77 ^***^	−0.03 (0.02)	0.97 [0.94–1.00]	**0.11 (0.02)**	1.12 [1.08–1.16]^*^	**0.15 (0.01)**	1.16 [1.14–1.19]^*^	**0.11 (0.02)**	1.11 [1.08–1.15]^*^
Gender (0 = female, 1 = male)	50.73 ^***^	−0.62 (0.40)	0.54 [0.25–1.18]	**1.09 (0.26)**	2.98 [1.80–4.92]^*^	**0.47 (0.23)**	1.61 [1.02–2.53]^*^	**−0.61 0.29)**	0.55 [0.31–0.96]^*^
Race (0 = minorities, 1 = white)	22.07 ^***^	**− 0.59 (0.28)**	1.82 [0.32–0.96]^*^	**−0.15 (0.35)**	0.86 [0.43–1.72]	− 0.56 (0.30)	0.57 [0.32–1.02]	**−1.05 (0.25)**	0.35 [0.22–0.56]^*^

*Note*: **χ**^**2**^ = chi-square independence (*df* = degrees of freedom); the Low Risk was the reference class; ^*^: *p* < .05; ^**^: *p* < .01; ^***^: *p* < .001

## Discussion

The goal of this analysis was to examine the multiple characteristics of comorbidity among CKD risk factors using a national probability sample of healthy middle and older adults in the United States. More than one-third of participants in this survey would be considered to be otherwise healthy and had low probabilities of evincing all risk factors. However, almost one-fifth of middle-aged and older adults were found to be members of the High Risk class, which was characterized by a high probability of evincing all the assessed risk factors. The rest of the participants met the criteria for being obese or having elevated BP or exhibiting the comorbidity of both obesity and elevated BP. Individuals in the Obese were more likely to have an elevated eGFR, whereas a comorbidity between obesity and elevated BP tended to be associated with a lower eGFR. In addition, the High Risk class was associated with the highest probability of having eGFR < 60 ml/min/1.73 m^2^. Finally, childhood SES was associated with class membership in this comorbidity of risk factors for CKD, independent of education level, current SES, age, gender, and race. Low childhood SES was significantly associated with a higher probability of being in the Obese and Elevated BP class and High Risk class rather than being in the healthier Low Risk class.

### Clustering of risk factors and eGFR

Among the five latent classes, the Obese class had the highest expected mean of eGFR, even higher than the Low Risk class, which may initially seem to be counterintuitive. However, there is an established association between obesity and hyperfiltration in the kidney. The mechanism behind kidney hyperfiltration among obese individuals is caused by vasodilation of kidney afferent arterioles and increased glomerular hydrostatic pressure [39, 40]. Vasodilation of kidney afferent arterioles among obese individuals is appears to be caused by dysregulation of the tubuloglomerular feedback (TGF) mechanism (see [7] for details) that controls the balance between sodium input and output. Dysregulation of the TGF mechanism in the kidney leads to increased sodium reabsorption in the tubules among obese individuals [39, 40]. Higher sodium reabsorptions in the tubules leads to a decrease sodium concentration at macula densa, causing kidney afferent arterioles vasodilation, which in turn will increase renal blood flow, GFR, and systemic blood pressure. Multiple mechanisms are also involved in the context of excessive tubular sodium reabsorptions among obese individuals, including [40, 41]: 1) kidney compression, 2) the overactivation of the renin-angiotensin-aldosterone system (RAAS), and 3) overactivation of kidney mineralocorticoid receptor (MR).

The alteration in the TGF mechanism due to an increase in sodium reabsorption at the kidney tubules may be adaptive at first, to achieve a balancing of sodium intake and output in the obese individuals. However, the effort to balance sodium is achieved at the cost of elevating blood pressure. Increased BP will eventually elevate glomerular hydrostatic pressure that will cause kidney damage [40, 41]. As indicated by our findings, membership in the Obese and Elevated BP class was associated with having the lowest expected mean of eGFR when compared to other classes. This difference is suggestive of a progressive decrement in kidney function over time as obese adults progress to chronically elevated BP, revealing the important age-related association between cardiovascular and renal physiology. A previous study found that obese individuals experienced a more rapid decrease in kidney function over time, especially in older individuals [42]. Hypertension and dyslipidemia are clinical warning signs for obese individuals that their kidney function will become compromised [43]. The eGFR for the majority of participants in the Obese and Elevated BP class would meet the clinical criterion for Stage 2 CKD. The MIDUS 2 participants are currently being reevaluated approximately 10 years after the prior assessment so there will be an opportunity to formally test if individuals from Obese class have transitioned to the Obese and Elevated BP class over time.

The High Risk class was the only category that had a high probability of also having an elevated glycosylated hemoglobin levels indicative of type 2 diabetes. While this class generally had a higher mean eGFR when compared to the Obese and Elevated BP class, membership in this class was associated with the highest probability of having a low eGFR indicative of Stage 3 CKD. The dual impact of central adiposity and insulin resistance in the High Risk adults would be in keeping with the view that obesity is a gateway condition [44] that precedes many chronic health conditions including diabetes, cardiovascular disease, and CKD. The Non-Obese but Elevated BP class also offers some distinctive insights because it reaffirms the important bidirectional relationship between renal clearance and blood pressure even in the absence of frank obesity. Membership in this class was characterized by a higher percentage of older participants. Given prior findings on the important influence of subclinical inflammatory activity [5], their lower kidney function may also be indicative of the contributory effects of cytokines and other factors that can dysregulate and accelerate the aging of the kidney. There has also been some discussion about whether the criterion for Stage 3 CKD should be modified in the elderly patient because some degree of renal decline is a normal part of aging [43].

### Childhood SES, risk factors, and kidney function

While several studies have previously documented the association between adult SES and risk factors for CKD [11], the current analysis demonstrates the important influence of childhood SES on the different characteristics of clustering among CKD risk factors. The significant influence of childhood SES, after controlling for education, current SES, age, gender, and race, indicated that the variances in the clustering of CKD risk factors was not completely explained by contemporaneous adult behavior and social standing. It reaffirms the importance of early life experiences as a critical period for establishing the developmental trajectory for health or disease in adulthood. While we do not have information regarding the socioeconomic conditions of their mothers during pregnancy and indicators of IUGR such as low birth weight or prematurity, the childhood socioeconomic measures that we used (e.g., parental education) would be in keeping with the view that adversity during pregnancy and the prenatal period would be linked to early life programming and postnatal disease outcomes later in adulthood. Parental education, for example, has been linked to factors associated with IUGR, including undernutrition/ malnutrition, maternal disease (e.g., hypertension, diabetes), and toxin exposure (e.g., smoking, alcohol, drugs) [20, 45–49].

### Limitations and future directions

Several limitations of this study should be acknowledged. First, our findings were based on cross-sectional data. Thus, all the results are purely associational, even though there was a temporal component to link early life experiences with adult health outcomes. Second, the information on childhood SES was based on retrospective self-report, which could introduce some recall bias. Although a separate examination of some participants who had siblings in the MIDUS project, including identical and fraternal twins, indicated a high concordance for the recall of childhood SES [50]. Third, the latent classes of CKD risk factors were treated similarly and as homogenous across age, sex, and racial groups. Given that each of these factors is known to influence kidney function, future analyses should formally test the heterogeneity of CKD risk factors within each subgroup. Although a large bias due to race would not be likely to have accounted for the overall conclusions given that both white and black Americans were represented in this study, it is likely that the magnitude of the associations could be different in other racial groups, such as Native Americans. For example, a prior analysis had shown that there are differences in the age-related decline in GFR between American and Japanese adults [5].

Another consideration is that only the CKD-EPI formula was used to calculate the eGFR. Previous analyses have indicated this formula works more optimally for capturing the later stages of declining CKD [5]. But the value of CKD-EPI formula for the primary aims of this analysis was that it takes age, sex, and race into consideration, addressing the caveat above about potential bias if conclusions were driven more by one subgroup of participants. Given our interest in the potential influence of obesity and central adiposity as an independent pathway of risk, we did not specifically correct for obesity when calculating the eGFR, even though it is possible there is an influence of adiposity on muscle mass, which could have affected serum creatinine levels. It is also important to consider that the modeling assumed some causal directionality in the association between the identified risk factors and kidney function. In reality, the linkages with kidney health are more complex as exemplified by a strong correlation between elevated BP and poorer renal clearance. Future research should also include other measures of glomerular status, and employ some of the administered substances that provide a more specific indication of clearance rates in a clinical setting. Finally, our approach to testing the association between childhood SES and class membership by considering education and adult SES as covariates could introduce some bias [51], as childhood SES, education, and adult SES are often strongly correlated. Future research should prioritize using a mediation approach to test whether childhood SES link to adult health reflects some of the limitations on upward mobility in the United States and thus engenders an invariant socioeconomic trajectory that is maintained into adulthood [14].

## Conclusions

We demonstrated that a 5-class model of risk optimally captures the variations of comorbidity among prominent risk factors for CKD, resulting in a taxonomy comprised of Low Risk, Obese, Obese and Elevated BP, Non-Obese but Elevated BP, and High Risk. Membership in the High Risk class was associated with a higher probability of having poorer kidney function (eGFR < 60 ml/min/1.73 m^2^). Conversely, the absence of the known risk factors can be considered protective and be indicative of more robust kidney function even into older adulthood. The most novel aspect of this analysis was our confirmation that latent class membership of comorbidity of risk factors was associated with childhood SES, even after controlling for education level, current SES, age, gender, and race, documenting the importance of early rearing conditions for the potential development of CKD in adulthood. A clearer understanding of health disparities requires consideration of both traditional clinical risk factors as well as the pervasive influence of sociodemographic processes that can accelerate and worsen the physiological changes and dysregulation associated with normal aging.

## Supplementary information

**Additional file 1: Supplemental Table.** Model Fit Information for Latent Class Analysis.

## Data Availability

The MIDUS datasets used in this analysis are available in the Inter-university Consortium for Political and Social Research (ICPSR) website: https://www.icpsr.umich.edu/icpsrweb/ICPSR/series/00203/studies?archive=ICPSR&sortBy=7

## Abbreviations

AIC: Akaike information criterion
BIC: Bayesian information criterion
a-BIC: sample size adjusted BIC
BLRT: Bootstrapped likelihood ratio test
BP: Blood pressure
CAIC: The “consistent AIC”
CI: Confidence interval
CKD: Chronic kidney disease
CKD-EPI: Chronic Kidney Disease Epidemiology Collaboration
CRP: C-reactive protein
CRU: Clinical research unit
DOHaD: Developmental origin of health and disease
eGFR: Estimated glomerular filtration rate
IL-6: Interleukin-6
IUGR: Intrauterine growth restriction
LCA: Latent class analysis
MIDUS: Midlife in the United States
MR: Mineralocorticoid receptor
RAAS: Renin-angiotensin-aldosterone system
SES: Socioeconomic status
TGF: Tubuloglomerular feedback

## References

[1] Levey AS, Coresh J (2012). Chronic kidney disease. Lancet.

[2] Levey AS, Stevens LA, Coresh J (2009). Conceptual model of CKD: applications and implications. Am J Kidney Dis.

[3] Haroun MK, Jaar BG, Hoffman SC, Comstock GW, Klag MJ, Coresh J (2003). Risk factors for chronic kidney disease: a prospective study of 23,534 men and women in Washington County, Maryland. J Am Soc Nephrol.

[4] Yamagata K, Ishida K, Sairenchi T, Takahashi H, Ohba S, Shiigai T, Narita M, Koyama A (2007). Risk factors for chronic kidney disease in a community-based population: a 10-year follow-up study. Kidney Int.

[5] Costello-White R, Ryff CD, Coe CL (2015). Aging and low-grade inflammation reduce renal function in middle-aged and older adults in Japan and the USA. Age (Dordr).

[6] Hall ME, do Carmo JM, da Silva AA, Juncos LA, Wang Z, Hall JE (2014). Int J Nephrol Renovasc Dis.

[7] Hall JE, do Carmo JM, da Silva AA, Wang Z, Hall ME (2019). Obesity, kidney dysfunction and hypertension: mechanistic links. Nat Rev Nephrol.

[8] Chagnac A, Herman M, Zingerman B, Erman A, Rozen-Zvi B, Hirsh J, Gafter U (2008). Obesity-induced glomerular hyperfiltration: its involvement in the pathogenesis of tubular sodium reabsorption. Nephrol Dial Transplant.

[9] Lynch J, Kaplan G (2000). Socioeconomic position, vol. 2000: Social epidemiology.

[10] Vart P, Gansevoort RT, Joosten MM, Bültmann U, Reijneveld SA (2015). Socioeconomic disparities in chronic kidney disease: a systematic review and meta-analysis. Am J Prev Med.

[11] Vart P, Gansevoort RT, Crews DC, Reijneveld SA, Bultmann U (2015). Mediators of the association between low socioeconomic status and chronic kidney disease in the United States. Am J Epidemiol.

[12] Ben-Shlomo Y, Kuh D. A life course approach to chronic disease epidemiology: conceptual models, empirical challenges and interdisciplinary perspectives. Int J Epidemiol. 2002;31(2):285–93. 11980781

[13] Pollitt RA, Rose KM, Kaufman JS (2005). Evaluating the evidence for models of life course socioeconomic factors and cardiovascular outcomes: a systematic review. BMC Public Health.

[14] Surachman A, Wardecker B, Chow SM, Almeida D (2019). Life course socioeconomic status, daily stressors, and daily well-being: examining chain of risk models. J Gerontol B Psychol Sci Soc Sci.

[15] Gruenewald TL, Karlamangla AS, Hu P, Stein-Merkin S, Crandall C, Koretz B, Seeman TE (2012). History of socioeconomic disadvantage and allostatic load in later life. Soc Sci Med.

[16] Surachman A, Rice C, Bray B, Gruenewald T, Almeida D (2020). Association between socioeconomic status mobility and inflammation markers among White and black adults in the United States: a latent class analysis. Psychosom Med.

[17] Brophy PD, Shoham DA, Charlton JR, Carmody JB, Reidy KJ, Harshman L, Segar J, D A (2015). Early-life course socioeconomic factors and chronic kidney disease. Adv Chronic Kidney Dis.

[18] Shoham DA, Vupputuri S, Kshirsagar AV (2005). Chronic kidney disease and life course socioeconomic status: a review. Adv Chronic Kidney Dis.

[19] Galobardes B, Smith GD, Lynch JW (2006). Systematic review of the influence of childhood socioeconomic circumstances on risk for cardiovascular disease in adulthood. Ann Epidemiol.

[20] Miller GE, Chen E, Parker KJ (2011). Psychological stress in childhood and susceptibility to the chronic diseases of aging: moving toward a model of behavioral and biological mechanisms. Psychol Bull.

[21] Laitinen J, Power C, Jarvelin MR (2001). Family social class, maternal body mass index, childhood body mass index, and age at menarche as predictors of adult obesity. Am J Clin Nutr.

[22] Parsons TJ, Power C, Logan S, Summerbell CD (1999). Childhood predictors of adult obesity: a systematic review. Int J Obes Relat Metab Disord.

[23] Lehman BJ, Taylor SE, Kiefe CI, Seeman TE (2009). Relationship of early life stress and psychological functioning to blood pressure in the CARDIA study. Health Psychol.

[24] Hogberg L, Cnattingius S, Lundholm C, Sparen P, Iliadou AN (2012). Intergenerational social mobility and the risk of hypertension. J Epidemiol Community Health.

[25] Lidfeldt J, Li TY, Hu FB, Manson JE, Kawachi I (2007). A prospective study of childhood and adult socioeconomic status and incidence of type 2 diabetes in women. Am J Epidemiol.

[26] Maty SC, Lynch JW, Raghunathan TE, Kaplan GA (2008). Childhood socioeconomic position, gender, adult body mass index, and incidence of type 2 diabetes mellitus over 34 years in the Alameda County study. Am J Public Health.

[27] Carroll JE, Cohen S, Marsland AL (2011). Early childhood socioeconomic status is associated with circulating interleukin-6 among mid-life adults. Brain Behav Immun.

[28] Stringhini S, Batty GD, Bovet P, Shipley MJ, Marmot MG, Kumari M, Tabak AG, Kivimaki M (2013). Association of lifecourse socioeconomic status with chronic inflammation and type 2 diabetes risk: the Whitehall II prospective cohort study. PLoS Med.

[29] Brim OG, Ryff CD, Kessler RC (2004). The MIDUS National Survey: an overview.

[30] Dienberg Love G, Seeman TE, Weinstein M, Ryff CD (2010). Bioindicators in the MIDUS national study: protocol, measures, sample, and comparative context. J Aging Health.

[31] Kirsch JA, Ryff CD (2016). Hardships of the great recession and health: understanding varieties of vulnerability. Health Psychol Open.

[32] Levey AS, Stevens LA (2010). Estimating GFR using the CKD epidemiology collaboration (CKD-EPI) Creatinine equation: more accurate GFR estimates, lower CKD prevalence estimates, and better risk predictions. Am J Kidney Dis.

[33] Tsenkova V, Pudrovska T, Karlamangla A (2014). Childhood socioeconomic disadvantage and prediabetes and diabetes in later life: a study of biopsychosocial pathways. Psychosom Med.

[34] Crandall CJ, Merkin SS, Seeman TE, Greendale GA, Binkley N, Karlamangla AS (2012). Socioeconomic status over the life-course and adult bone mineral density: the midlife in the U.S. study. Bone.

[35] Tsenkova VK, Lee C, Boylan JM (2017). Childhood socioeconomic disadvantage, occupational, leisure-time, and household physical activity, and diabetes in adulthood. J Phys Act Health.

[36] Lanza ST, Collins LM, Lemmon DR, Schafer JL (2007). PROC LCA: a SAS procedure for latent class analysis. Struct Equ Modeling.

[37] Bakk Z, Vermunt JK (2016). Robustness of Stepwise Latent Class Modeling With Continuous Distal Outcomes. Struct Equ Modeling.

[38] Dziak JJ, Bray BC, Wagner AT (2017). LCA_Distal_BCH SAS Macro Users’ Guide (Version 1.1).

[39] Hall JE, Crook ED, Jones DW, Wofford MR, Dubbert PM (2002). Mechanisms of obesity-associated cardiovascular and renal disease. Am J Med Sci.

[40] Hall JE, do Carmo JM, da Silva AA, Wang Z, Hall ME. Obesity, kidney dysfunction and hypertension: mechanistic links. Nat Rev Nephrol. 2019;15(6):367–85. 10.1038/s41581-019-0145. 10.1038/s41581-019-0145-4 PMC7278043 31015582

[41] Hall JE, Kuo JJ, da Silva AA, de Paula RB, Liu J, Tallam L (2003). Obesity-associated hypertension and kidney disease. Curr Opin Nephrol Hypertens.

[42] de Boer IH, Katz R, Fried LF, Ix JH, Luchsinger J, Sarnak MJ, Shlipak MG, Siscovick DS, Kestenbaum B (2009). Obesity and change in estimated GFR among older adults. Am J Kidney Dis.

[43] Denic A, Glassock RJ, Rule AD (2016). Structural and functional changes with the aging kidney. Adv Chronic Kidney Dis.

[44] Medina-Gomez G, Vidal-Puig A (2005). Gateway to the metabolic syndrome. Nat Med.

[45] Parker JD, Schoendorf KC, Kiely JL (1994). Associations between measures of socioeconomic status and low birth weight, small for gestational age, and premature delivery in the United States. Ann Epidemiol.

[46] Martinson ML, Reichman NE (2016). Socioeconomic inequalities in low birth weight in the United States, the United Kingdom, Canada, and Australia. Am J Public Health.

[47] Kramer MS (1998). Socioeconomic determinants of intrauterine growth retardation. Eur J Clin Nutr.

[48] Hendrix N, Berghella V. Non-placental causes of intrauterine growth restriction: Elsevier; 2008. p. 161–5. 10.1053/j.semperi.2008.02.004 18482615

[49] Kett MM, Denton KM (2010). Renal programming: cause for concern?. Am J Physiol Regul Integr Comp Physiol.

[50] Ward MM (2011). Concordance of sibling's recall of measures of childhood socioeconomic position. BMC Med Res Methodol.

[51] VanderWeele TJ, Hernan MA, Robins JM (2008). Causal directed acyclic graphs and the direction of unmeasured confounding bias. Epidemiology.

