# Supplementary material for: Childhood socioeconomic status, comorbidity of chronic kidney disease risk factors, and kidney function among adults in the midlife in the United States (MIDUS) study
Source: BMC Nephrol. 2020 May 19;21:188. doi: 10.1186/s12882-020-01846-1 (PMC7236129; doi:10.1186/s12882-020-01846-1)
Supplement: Supplementary file 1 — Additional file 1: Supplemental Table. Model Fit Information for Latent Class Analysis. [file 12882_2020_1846_MOESM1_ESM.docx]

Supplemental Material

Supplemental Table 1

Model Fit Information for Latent Class Analysis

| No. of classes | Log-likelihood | Degrees of freedom | AIC | BIC | CAIC | a-BIC | Entropy | BLRT |
| --- | --- | --- | --- | --- | --- | --- | --- | --- |
| 1 | -9368.58 | 120 | 2252.38 | 2266.38 | 2305.99 | 2283.75 |  |  |
| 2 | -8475.15 | 112 | 495.52 | 580.39 | 595.39 | 532.74 | .82 | *p* < .05 |
| 3 | -8391.03 | 104 | 343.28 | 473.42 | 496.42 | 400.34 | .73 | *p* < .05 |
| 4 | -8335.72 | 96 | 248.66 | 424.07 | 455.07 | 325.58 | .71 | *p* < .05 |
| **5** | **-8306.75** | **88** | **206.72** | **427.39** | **466.39** | **303.48** | **.69** | ***p* < .05** |
| 6 | -8289.67 | 80 | 188.57 | 454.50 | 501.50 | 305.18 | .67 | *p* > .05 |

*Note*: boldface type indicates selected model.

**AIC**: Akaike information criterion; **BIC**: Bayesian information criterion; **a-BIC**: sample size adjusted BIC; **BLRT** = bootstrapped likelihood ratio test; **CAIC**: the “consistent AIC”
